# Accuracy of microRNAs as markers for the detection of neck lymph node metastases in patients with head and neck squamous cell carcinoma

**DOI:** 10.1186/s12916-015-0350-3

**Published:** 2015-05-09

**Authors:** Ana Carolina de Carvalho, Cristovam Scapulatempo-Neto, Danielle Calheiros Campelo Maia, Adriane Feijó Evangelista, Mariana Andozia Morini, André Lopes Carvalho, André Luiz Vettore

**Affiliations:** Laboratory of Cancer Molecular Biology, Department of Biological Sciences, Diadema Campus, Federal University of São Paulo, Rua Pedro de Toledo, 669, São Paulo, SP 04039-032 Brazil; Molecular Oncology Research Center, Barretos Cancer Hospital, Rua Antenor Duarte Vilela, 1331, Barretos, SP 14784-400 Brazil; Department of Pathology, Barretos Cancer Hospital, Rua Antenor Duarte Vilela, 1331, Barretos, SP 14784-400 Brazil; Department of Head and Neck Surgery, Barretos Cancer Hospital, Rua Antenor Duarte Vilela, 1331, Barretos, SP 14784-400 Brazil; Cancer Stem Cell Biology Program, Duke-NUS Graduate Medical School, 8 College Road, Singapore, 169857 Singapore

**Keywords:** Diagnostic markers, Fine-needle aspiration biopsies, Head and neck cancers, MicroRNAs, miR-200 family, miR-203, miR-205, Neck metastases

## Abstract

**Background:**

The presence of metastatic disease in cervical lymph nodes of head and neck squamous cell carcinoma (HNSCC) patients is a very important determinant in therapy choice and prognosis, with great impact in overall survival. Frequently, routine lymph node staging cannot detect occult metastases and the post-surgical histologic evaluation of resected lymph nodes is not sensitive in detecting small metastatic deposits. Molecular markers based on tissue-specific microRNA expression are alternative accurate diagnostic markers. Herein, we evaluated the feasibility of using the expression of microRNAs to detect metastatic cells in formalin-fixed paraffin-embedded (FFPE) lymph nodes and in fine-needle aspiration (FNA) biopsies of HNSCC patients.

**Methods:**

An initial screening compared the expression of 667 microRNAs in a discovery set comprised by metastatic and non-metastatic lymph nodes from HNSCC patients. The most differentially expressed microRNAs were validated by qRT-PCR in two independent cohorts: i) 48 FFPE lymph node samples, and ii) 113 FNA lymph node biopsies. The accuracy of the markers in identifying metastatic samples was assessed through the analysis of sensitivity, specificity, accuracy, negative predictive value, positive predictive value, and area under the curve values.

**Results:**

Seven microRNAs highly expressed in metastatic lymph nodes from the discovery set were validated in FFPE lymph node samples. MiR-203 and miR-205 identified all metastatic samples, regardless of the size of the metastatic deposit. Additionally, these markers also showed high accuracy when FNA samples were examined.

**Conclusions:**

The high accuracy of miR-203 and miR-205 warrant these microRNAs as diagnostic markers of neck metastases in HNSCC. These can be evaluated in entire lymph nodes and in FNA biopsies collected at different time-points such as pre-treatment samples, intraoperative sentinel node biopsy, and during patient follow-up. These markers can be useful in a clinical setting in the management of HNSCC patients from initial disease staging and therapy planning to patient surveillance.

**Electronic supplementary material:**

The online version of this article (doi:10.1186/s12916-015-0350-3) contains supplementary material, which is available to authorized users.

## Background

Head and neck squamous cell carcinoma (HNSCC) is one of the most common cancers in the world, with an incidence of approximately 600,000 new cases per year [1]. Despite different strategies employed in the treatment of HNSCC patients, including surgery, radiotherapy, and chemotherapy, late diagnosis and the frequent development of loco-regional recurrences frequently contribute to treatment failure and low rates of overall survival observed [2].

The involvement of neck lymph nodes in HNSCC patients significantly reduces the odds for disease control. The 5-year survival rate for patients without lymph node metastases is 63% to 86%, while patients with neck metastases have rates of 20% to 36% [3-6]. Besides being an important prognostic indicator, the presence of metastatic disease in lymph nodes also influences the choice of the adjuvant therapy used to reduce disease recurrence [7-9]. Therefore, assessment of the neck has become an integral part of the treatment planning for HNSCC patients.

There is a well-accepted strategy for the treatment of patients with clinically positive lymph nodes, which includes therapeutic neck dissection followed by postoperative radiotherapy with or without concurrent chemotherapy. However, the treatment choice for patients without clinical evidence of neck metastases remains controversial [10,11]. Initial tumors are highly curable by surgery or radiotherapy alone. For this reason, a ‘wait-and-see’ approach, in which the neck is not treated and the patient is followed and monitored with special attention to the evolution of disease in cervical lymph nodes is acceptable. Nevertheless, studies have shown that 30% to 60% of cases with initial tumors will present cervical metastases [12-14]. On the other hand, the use of elective neck dissection for all patients with early tumors and clinical and radiological N0 necks at high risk of harboring subclinical disease in the lymph nodes (mainly tongue and floor of the mouth sites, T2 tumors with high depth of invasion) is also defended [15]. Although some studies showed a better loco-regional control and longer regional disease-free survival for patients submitted to elective neck dissection [16,17], only 20% to 50% will actually harbor metastatic disease in their lymph nodes. In other words, 50% to 80% of these patients were subjected to the morbidity of unnecessary surgical treatment [12-14,16,18].

A limitation for the correct evaluation of neck metastases in HNSCC patients is the lack of sufficient sensitivity of the postoperative assessment of lymph nodes by formalin-fixed tissue sections stained with hematoxylin and eosin (H&E), especially regarding small metastatic deposits. Studies show that 10% to 40% of HNSCC patients with histopathologically negative neck lymph nodes eventually develop regional metastases, suggesting that metastatic cells present in the lymph nodes could not be detected at diagnosis [12,19,20]. In line with this, 5% to 20% of lymph nodes previously classified as metastasis-free by routine histopathological evaluation present positivity for cytokeratins in immunohistochemical (IHC) analysis [21-25]. To overcome this problem, molecular detection of metastases seems to be one of the most promising methods for definitive lymph node evaluation. Through this approach, metastatic deposits in lymph nodes can be detected in a more sensitive, accurate, and less time-consuming manner. Toward this end, the identification of molecular markers capable of detecting the presence of metastatic cells in a background of lymphatic cells is mandatory.

MicroRNAs are small non-coding RNA molecules of approximately 22 nucleotides, described as regulators of gene expression in a variety of multicellular organisms [26,27]. These small molecules interact mainly with the 3′-untranslated regions of specific messenger RNAs (mRNA), inducing their degradation or inhibiting their translation [26,27]. Specific microRNA expression has been observed in different cancer types and at distinct differentiation stages [28-31]. These molecules also play an important role in the development of head and neck cancers. HNSCC-specific microRNA expression profiles and key microRNAs known to orchestrate gene and protein expression levels in these tumors have been established [32]. Recent studies have highlighted different applications of these molecules as biomarkers in HNSCC, such as in the detection of HNSCC cells in saliva samples, in the identification of human papilloma virus-positive oropharyngeal tumors, or as prognostic markers associated with disease progression [32-36]. Furthermore, Fletcher et al. [37] found that the expression of miR-205 was specific to metastatic lymph nodes of HNSCC patients.

In this article, we report the identification of microRNAs capable of detecting metastatic cells in formalin-fixed paraffin-embedded (FFPE) lymph nodes and in fine-needle aspiration (FNA) biopsies of HNSCC patients with high sensitivity, specificity, and accuracy.

## Methods

### Population cohorts

The current study included retrospective and prospective cohorts. The retrospective cohort involved FFPE neck lymph node samples from 48 patients surgically treated between 2000 and 2012 at the Department of Head and Neck Surgery, Barretos Cancer Hospital, Barretos, SP, Brazil. The inclusion criteria of this cohort were patients with primary SCC of the oral tongue, floor of mouth, lower gum, and retromolar area; classified as T1-T2-T3 stages and without clinical and radiological evidence of metastases in cervical lymph nodes at diagnosis; submitted to surgery as the first therapeutic modality for treatment of the primary tumor; plus elective supraomohyoid neck dissection or sentinel lymph node evaluation. The use of these samples was approved by the Barretos Cancer Hospital Institutional Review Board.

The prospective cohort was comprised by FNA biopsies of resected lymph nodes from 79 patients who received treatment between 2013 and 2014 at the Department of Head and Neck Surgery of the Barretos Cancer Hospital, Barretos, SP, Brazil. This cohort included HNSCC patients, regardless of tumor site or stage, who underwent neck dissection during the surgery of the primary tumor, as a salvage treatment after organ preservation protocol, or for the treatment of patients who developed neck metastases during follow-up. Informed consent was obtained from each individual prior to tissue collection and the study protocol was approved by Barretos Cancer Hospital Institutional Review Board.

### Study design

This study can be divided into three distinct phases. Firstly, a ‘discovery set’ comprising metastatic and non-metastatic FFPE lymph nodes from patients with tumors in the oral cavity was evaluated in order to select promising markers to be tested in a larger sample cohort. This analysis allowed the selection of good candidates to be evaluated in a ‘validation set’ containing an additional group of metastatic and non-metastatic FFPE lymph nodes from patients presenting oral cavity tumors. This analysis identified microRNAs with high sensitivity and specificity in detecting the presence of metastatic cells in lymph nodes. Later, the accuracy of these markers was evaluated in an independent prospective cohort of FNA samples collected from negative and positive lymph nodes from HNSCC patients harboring tumors in the oral cavity, pharynx, and larynx.

### FFPE samples processing and RNA purification

H&E sections corresponding to paraffin blocks containing the samples of interest were reviewed by a pathologist to confirm the diagnosis and for characterization of the cellular components present in the samples.

**Figure 1:**
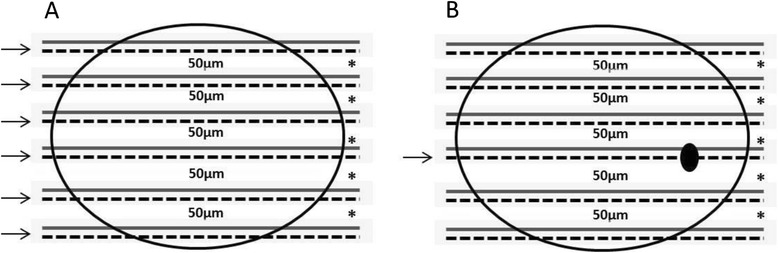
Schematic representation of lymph nodes sectioning protocol for immunohistochemistry (solid line) and molecular (dotted line) analyses. **(A)** Non-metastatic lymph nodes: all sections at different levels were used for RNA extraction and subsequent molecular analysis (arrows). **(B)** Metastatic lymph nodes: only sections of the positive level at IHC analysis were used for RNA extraction and subsequent molecular analysis (arrow). The leftover material (regions marked with “*”) removed from each interval of 50 μm was collected and used in the analysis of the leftover material.

To confirm the absence of metastatic deposits in histologically free-of-metastases cases, the blocks containing all lymph nodes resected from each patient were step-sectioned at 50 μm intervals and six sections with a thickness of 5 μm each were obtained from each level (Figure 1). The first section was examined by IHC analysis for the expression of cytokeratins, while the remaining five sections were used in molecular analyses. Samples were finally classified as negative when the specimen contained no tumor cells (no positivity for cytokeratins) and positive when isolated tumor cells (<0.2 mm), micrometastasis (0.2–2.0 mm), or macrometastasis (>2.0 mm) were detected.

We also challenged the selected markers to detect the presence of metastatic cells in the leftover material removed from each level (intervals of 50 μm) of the step-sectioned FFPE lymph nodes examined in the IHC analyses (Figure 1).

For RNA extraction from FFPE positive lymph node cases, macrodissection was applied to five paraffin-embedded sections to collect the metastatic deposit prior to RNA extraction. For the non-metastatic FFPE cases, total RNA was extracted from sections obtained from different levels of the lymph nodes. Total RNA was isolated using the Recoverall Total Nucleic Acid Isolation kit (Ambion, Austin, TX, USA), which contains a DNAse treatment step. Total RNA was quantified in the Qubit fluorometer (Invitrogen, Carlsbad, CA, USA) and stored at −80°C until use.

Due to the scarcity of RNA quantity yielded from FFPE samples, no step for evaluation of the RNA purity or integrity was performed. The amplification of the internal controls (U6 and U47) by qPCR was used as indicative of the high quality of the RNA samples. It is important to mention that all samples included in the study showed amplification of both internal controls at early cycles.

### Fine-needle aspiration biopsies processing and RNA purification

The material collected through FNA of the resected lymph nodes was used to perform a smear, and the slides were stained to enable the cytological diagnostic of the lymph nodes as ‘positive’ or ‘negative’ for the presence of metastatic cells. The remaining material on the needle was washed in 200 μL of sterile saline solution and ethylenediaminetetraacetic acid, snap frozen, and stored at −80°C until RNA extraction.

The extraction of total RNA from FNA samples was performed using the Trizol reagent (Invitrogen, Carlsbad, CA, USA) as previously described [38]. Total RNA was quantified in the Qubit fluorometer (Invitrogen) and stored at −80°C until use.

### Global microRNA profiling by TaqMan human MicroRNA arrays

In the ‘discovery set’, a global microRNA expression profiling was performed in lymph nodes from six FFPE oral tongue SCC samples (four with macrometastases and two with non-metastatic nodes) using TaqMan Human MicroRNA Arrays (Applied Biosystems, Foster City, CA, USA).

Total RNA (40 ng) from each lymph node sample were reverse transcribed into cDNA using the TaqMan microRNA Kit and Megaplex RT Primers (both from Applied Biosystems). After synthesis, the cDNA was pre-amplified using the TaqMan PreAmp Master Mix Kit and Megaplex PreAmp Primers (Applied Biosystems). The amplified-cDNA was then transferred to the TaqMan Human MicroRNA Array plates and the amplification was carried out in an Applied Biosystems 7900HT Real-Time PCR system.

The data obtained was analyzed using the software DataAssist v3.0 (Applied Biosystems). The fold-change difference between metastatic and non-metastatic lymph node samples was calculated using the 2^-ΔΔCt^ method [39]. The small nuclear RNA U6 was used as an endogenous control and the non-metastatic group was assigned as a reference since this was the most stable control in the assay.

### Validation of differentially expressed microRNAs by real-time PCR

The ‘validation set’ included 48 FFPE lymph node samples and 113 FNA lymph node biopsies from HNSCC patients. Each assay was conducted using the Taqman MicroRNA Reverse Transcription kit (Applied Biosystems) according to the manufacturer’s protocols. Briefly, 10 ng of total RNA was reverse-transcribed to cDNA using a MultiScribe Reverse Transcriptase and a stem-loop primer (Applied Biosystems) specific for each selected microRNA according to manufacturer’s instructions. Quantitative real-time PCR (qRT-PCR) was performed using a TaqMan PCR kit on a 7500 Fast Real-Time PCR System (Applied Biosystems). All reactions were performed in triplicate. To evaluate the differential expression of each microRNA between metastatic and non-metastatic lymph nodes, the 2^-ΔΔCt^ method was employed [39]. The ratio between the average Ct values of each microRNA and the internal controls (U6 and U47) in 10 non-metastatic lymph node samples was used as reference in the 2^-ΔΔCt^ formula.

### Statistical analysis

In the discovery set, to search for differentially expressed microRNAs in metastatic and non-metastatic lymph nodes, a global microRNA expression profiling was conducted. In this analysis, ΔCt values from each microRNA were evaluated by the non-parametric Rank Products test using the RankProd R package [40]. The Rank Products test is a robust tool to perform ranking lists with high performance on biological validation. This method did not give any assumption on the data distribution, but provided frequency-based ranking scores. In the validation sets (FFPE and FNA), differentially expressed microRNAs were identified using the Mann–Whitney U test.

A receiver operating characteristic (ROC) curve was constructed with the expression levels of each microRNA of interest in the metastatic and non-metastatic samples. The ROC curve is a plot of sensitivity (Se) versus 1-specificity (1-Sp) at all possible expression levels (c). In the FFPE validation set, a cutoff value was determined for each individual marker to maximize the classification accuracy according to the Youden index. The Youden Index (J) is a way to summarize ROC curve statistics in the interpretation and evaluation of a biomarker. It defines the maximum potential effectiveness of a biomarker and can be formally defined as J = max c {Se(c) + Sp (c) − 1} (value in which the difference between sensitivity and 1-specificity is maximum) [41]. For the FNA validation set, a 10-fold cutoff level was adopted to simplify the analysis and allow better reproducibility of the tests.

The area under the ROC curve (AUC) was able to identify optimal sensitivity and specificity levels to distinguish metastatic samples from non-metastatic ones. Sensitivity, specificity, accuracy, and positive and negative predictive values (PPV and NPV) of each individual microRNA in distinguishing metastatic from non-metastatic samples were also calculated along with 95% confidence intervals (95% CI). For FNA samples, the agreement between molecular findings with cytological and histological diagnostic was assessed using the Kappa test.

All two-tailed *P* values were derived from statistical tests, using a computer-assisted program (IBM SPSS Statistics, Version 19), and considered statistically significant at *P* <0.05.

## Results

### Patient characteristics

Clinical and histopathological data of the patients enrolled in this study are presented in Additional file 1: Tables S1 and S2.

For the FFPE series, of the 48 patients profiled in this cohort, 47.9% were smokers, 79.2% were males, and the age ranged from 43 to 84 years (median 60 years). Primary tumor sites were oral tongue (58.3%), floor of mouth (31.3%), alveolar ridge (8.3%), and lower gum (2.1%) and most were cT2 (62.5%). All patients in this cohort underwent surgery as the primary modality of treatment, and 23 (47.9%) received adjuvant radiation or chemo-radiation therapy.

For the FNA cohort, of the 79 patients included in this cohort, 76% were smokers and 88.6% were males, with age ranging from 29 to 78 years (median 57 years). The primary tumor sites were oral cavity (59.5%), oropharynx (19.0%), larynx (15.2%), and hypopharynx (6.3%) and 78.5% had advanced disease (III–IV). All patients in this cohort underwent neck dissection either during the surgery of the primary tumor (69.6%), as a salvage treatment after organ preservation protocol (24.1%), or for the treatment of patients who developed neck metastases during follow-up (6.3%).

### Identification of metastatic cell deposits in FFPE and FNA lymph node samples

**Figure 2:**
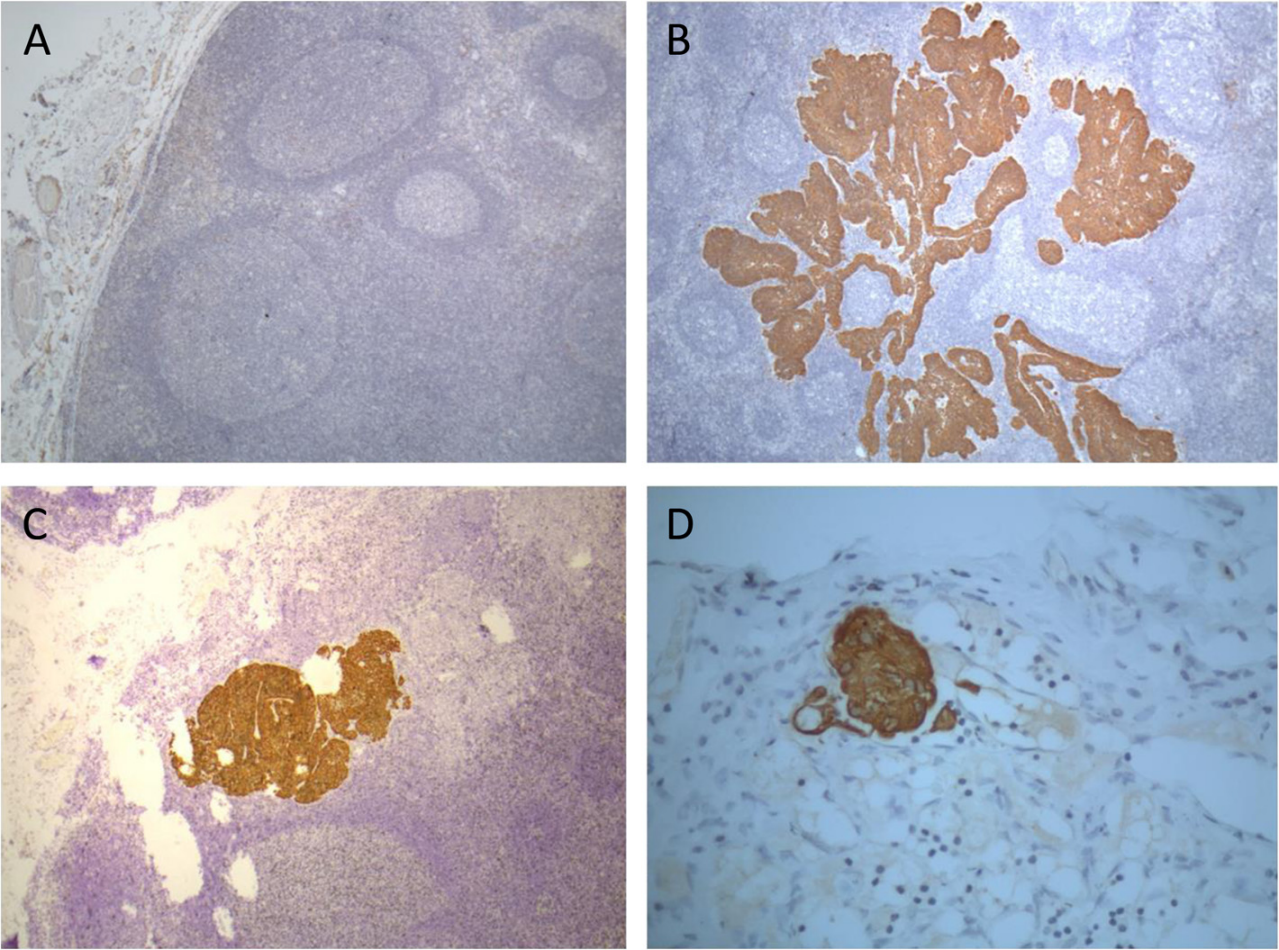
Immunohistochemistry staining for cytokeratins (M3515, clone AE1/AE3, Dako) in histologically negative lymph nodes of HNSCC patients. **(A)** Lymph node without evidence of metastases. **(B)** Lymph node with macrometastases. **(C)** Lymph node with micrometastases. **(D)** Lymph node with isolated tumor cells. **A**, **B**, and **C**: 40× magnification; **D**: 400× magnification.

A total of 356 lymph nodes resected from the 48 patients included in the FFPE cohort were examined through H&E to provide the histologic diagnostic of the lymph nodes for the presence of metastatic cells. All histologically-free of metastases lymph nodes were further step-sectioned and submitted to IHC for cytokeratins to confirm the absence of metastatic cells and to identify possible small metastatic deposits. Therefore, of the 48 patients included in the FFPE cohort, 25 harbored metastatic lymph nodes (18 with macrometastases, 5 with micrometastases, and 2 with isolated tumor cells) and 23 samples had metastases-free lymph nodes (Figure 2).

Overall, 113 FNA biopsies were collected from lymph nodes resected from 79 patients submitted to neck dissection. During the collection, whenever possible, FNA biopsies were conducted in macroscopically positive and negative lymph nodes from the same patient. These samples were further classified as ‘positive’ or ‘negative’ according to the cytological examination of the lymph node biopsies. Moreover, the resected lymph nodes were processed according to the routine of the Department of Pathology and H&E sections were assessed to provide the histologic diagnostic of the lymph nodes as ‘positive’ or ‘negative’ for the presence of metastatic cells. For three of the 113 FNA samples, there was a disagreement between histological and cytological diagnostics. While the cytological smears were classified as negative in all three samples, the histological assessment found micrometastatic deposits in two cases and isolated tumor cells in the third one. Thus, according to the cytological evaluation, 42 (37.2%) of the FNA samples collected were classified as positive and 71 (62.8%) as negative; in the histological evaluation, 45 (39.8%) were classified as positive and 68 (60.2%) were classified as negative for the presence of metastatic epithelial cells.

### Global microRNA profiling in lymph node samples

**Figure 3:**
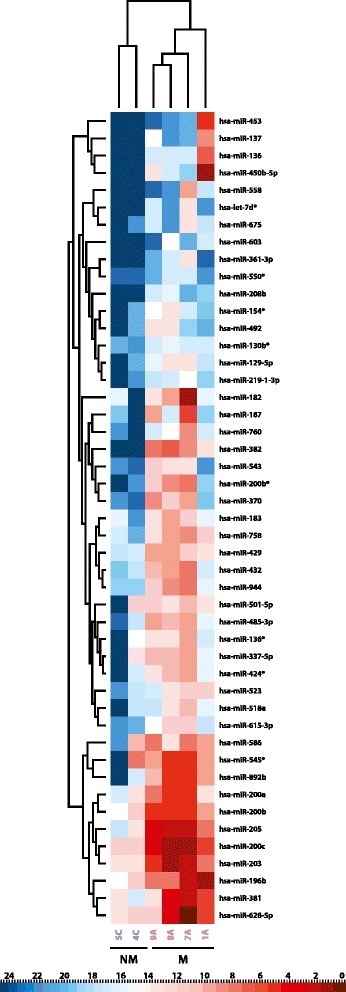
Heatmap representations of the 47 differentially expressed microRNAs with fold-change ≥2 and *P* value <0.05 (Rank Products) in the comparison between four metastatic lymph nodes (M: 7A, 1A, 8A, and 9A) and two non-metastatic lymph nodes (NM: 4C and 5C) resected from patients with T2N0 tongue squamous cell carcinomas. Non-supervised hierarchical clustering plotted based on the average ΔCt values. Up-regulated and down-regulated microRNAs are shown as red and blue, respectively. The columns represent samples and the microRNAs differentially expressed are shown in the lines.

Comprehensive microRNA profiles were generated for metastatic (n = 4) and non-metastatic (n = 2) lymph nodes from HNSCC patients using a quantitative RT-PCR array platform. From a total of 667 microRNAs, 439 were detected in at least two samples, regardless of the group, thereby serving as the pool of data for further analyses. From those, 61 presented a *P* <0.05 in the Rank Products test with 47 showing at least two-fold upregulation in all four metastatic samples. A non-supervised hierarchical clustering analysis using the ΔCt values of these 47 microRNAs displayed two distinct clusters formed by metastatic and non-metastatic lymph nodes (Figure 3). Finally, seven microRNAs (miR-200a, miR-200c, miR-203, miR-205, miR-382, miR-628-5p, miR-758), presenting more than 100-fold increment in the expression level in metastatic lymph nodes, were selected for further analyses (Table 1).

**Table 1:** **Seven microRNAs highly upregulated in metastatic lymph node samples collected from HNSCC patients according to the global microRNA profiling using the TaqMan human microRNA array cards**

**microRNAs**	**Mean fold-change**	***P*** value*
miR-200a	594	0.008
miR-200c	628	0.006
miR-203	2169	0.001
miR-205	1648	0.0001
miR-382	82984	<0.00001
miR-628-5p	1564	0.01
miR-758	182	0.01

*Rank products.

### Evaluation of the differentially expressed microRNAs in FFPE samples

Due to the scarcity of RNA quantity recovery from the FFPE samples, the evaluation of the expression profile of the selected microRNAs in the FFPE ‘validation set’ was performed in two stages. In the first series, the expression level of the seven microRNAs selected in the ‘discovery set’ (miR-200a, miR-200c, miR-203, miR-205, miR-382, miR-628-5p, and miR-758) was examined in 19 positive lymph nodes (14 macrometastases and 5 micrometastases) and in 13 negative lymph nodes (of the 23 patients with negative lymph nodes included in this study, 10 were used as a control reference, as indicated in Methods section).

This analysis showed that miR-628-5p, miR-758, and miR-382, were only able to detect 26.3%, 31.6%, and 52.6% of the metastatic samples, respectively, reflecting a low sensitivity (Additional file 1: Table S3, Additional file 2: Figure S1) and were excluded from the study. On the other hand, miR-200a, miR-200c, miR-203, and miR-205 presented maximum specificity (100%) and high sensitivity (84.2%, 94.7%, 100%, and 100%, respectively) (Additional file 1: Table S3). Therefore, in the second series, these four microRNAs were tested in the expanded cohort of samples (25 metastatic and 23 non-metastatic lymph nodes).

**Figure 4:**
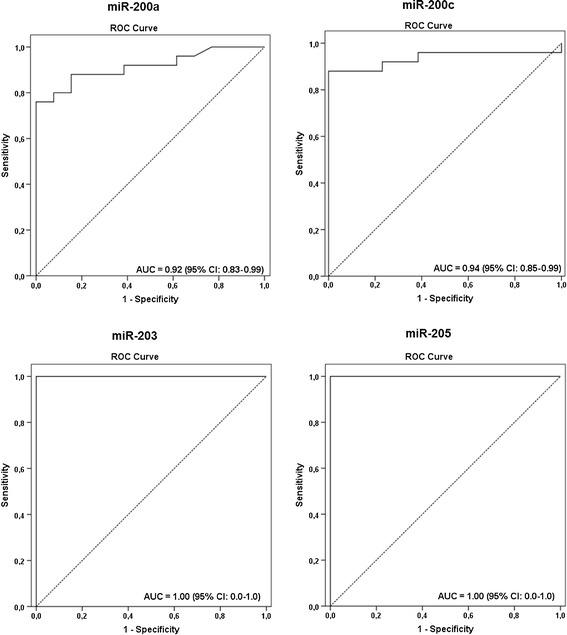
ROC (Receiver Operating Characteristic) curves obtained for microRNAs miR-200a, miR-200c, miR-203, and miR-205 in the FFPE validation set.

**Figure 5:**
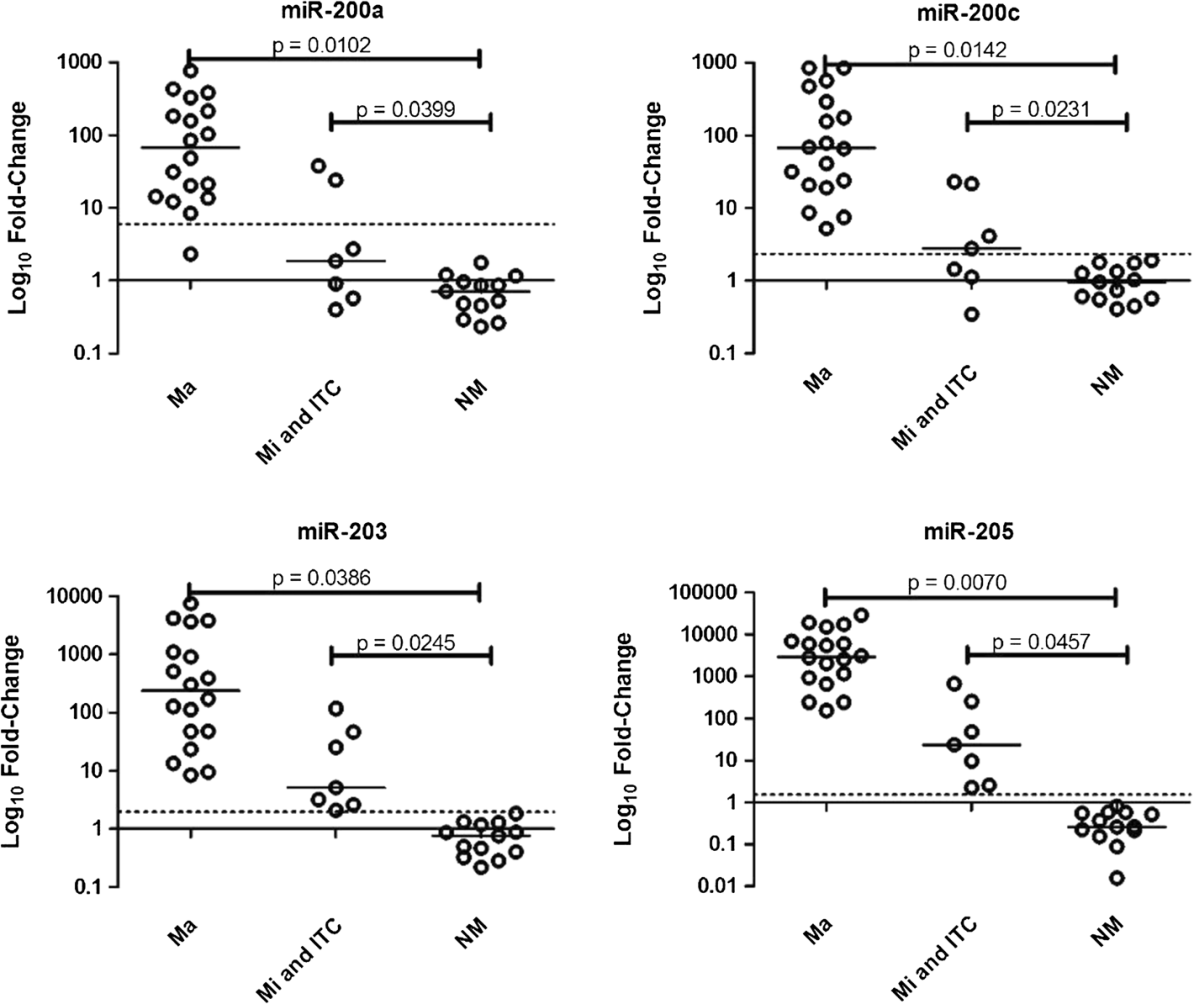
Expression profile of miR-200a, miR-200c, miR-203, and miR-205 in lymph node samples containing macrometastases (Ma; n = 18), micrometastases/isolated tumor cells (Mi and isolated tumor cells; n = 7), or in non-metastatic lymph node specimens (NM; n = 13). The Y-axis shows the log_10_ fold-change of the relative expression (2^-ΔΔCt^). The *P* value (Mann–Whitney) from each comparison is provided. The dotted line indicates the cutoff adopted according to the Youden index (value in which the difference between sensitivity and 1-specificity is maximum) obtained from the ROC curves analysis. The horizontal line indicated the median of fold-change values for each group.

The Youden index obtained from ROC curves was used to determine the cutoff values for upregulation of miR-200a, miR-200c, miR-203, and miR-205, which varied from 1.54 to 5.96 (Table 2, Figure 4). The expression profile of these four microRNAs in the metastatic and non-metastatic FFPE lymph node samples showed they were highly associated with the presence of metastatic cells in the neck lymph nodes of HNSCC cases (miR-200a, 76%; miR-200c, 88%; miR-203, 100%; miR-205, 100%; Figure 5, Table 2). However, miR-200a and miR-200c barely detected the presence of micrometastases (40% and 80%, respectively), and failed in detecting cases with isolated tumor cells. On the other hand, miR-203 and miR-205 displayed high sensitivity levels (100%) and were also able to correctly classify lymph nodes containing macrometastases, as well as micrometastases or isolated tumor cells (Table 2).

**Table 2:** **Sensitivity and specificity values of microRNAs evaluated in discriminating metastatic and non-metastatic lymph nodes in FFPE and FNA biopsies from lymph node samples**

**microRNA**		**Sensitivity**				**Specificity**
	**Cutoff** ^**a**^	**Metastatic** ^**b**^	**Macrometastases**	**Micrometastases**	**Isolated tumor cells**	**Non-metastatic**
		**% (95% CI) (n)**	**% (95% CI) (n)**	**% (95% CI) (n)**	**% (95% CI) (n)**	**% (95% CI) (n)**
**FFPE samples**						
**miR-200a**	5.96	76.0 (54.5–89.8) (19/25)	94.4 (70.6–99.7) (17/18)	40.0 (7.3–82.9) (2/5)	0 (0–80.2) (0/2)	100 (71.7–100) (13/13)
**miR-200c**	2.33	88.0 (67.7–96.8) (22/25)	100 (78.1–100) (17/18)	80.0 (29.9–98.9) (4/5)	0 (0–80.2) (0/2)	100 (71.7–100)) (13/13)
**miR-203**	1.96	100 (83.4–100) (25/25)	100 (78.1–100) (17/18)	100 (46.3–100) (5/5)	100 (19.8–100) (2/2)	100 (71.7–100) (13/13)
**miR-205**	1.54	100 (83.4–100) (25/25)	100 (78.1–100) (17/18)	100 (46.3–100) (5/5)	100 (19.8–100) (2/2)	100 (71.7–100) (13/13)
**FNA samples classified by cytology**						
**miR-203**	10	100 (91.5–100) (42/42)	100 (91.5–100) (42/42)	N/A	N/A	100 (94.9–100) (71/71)
**miR-205**	10	100 (91.5–100) (42/42)	100 (91.5–100) (42/42)	N/A	N/A	100 (94.9–100) (71/71)
**FNA samples classified by histology**						
**miR-203**	10	92.9 (80.5–98.4) (68/71)	100 (89.3–100) (68/68)	0 (0) (0/2)	0 (0) (0/1)	100 (94.9–100) (71/71)
**miR-205**	10	92.9 (80.5–98.4) (68/71)	100 (89.3–100) (68/68)	0 (0) (0/2)	0 (0) (0/1)	100 (94.9–100) (71/71)

FFPE, Formalin-fixed paraffin embedded; FNA, Fine-needle aspiration; PPV, Positive predictive value; NPV, Negative predictive value; AUC, Area under the ROC curve; CI, Confidence interval; N/A, Not applicable. ^a^The cutoff values for FFPE samples were determined according to the Youden index (value in which the difference between sensitivity and 1-specificity is maximum) obtained from the ROC curves. ^b^The ‘metastatic’ group comprises all cases with positive lymph nodes (macrometastases, micrometastases, or isolated tumor cells).

### Diagnostic accuracy of microRNAs in FFPE lymph node samples

The diagnostic accuracy of miR-200a, miR-200c, miR-203, and miR-205 in differentiating lymph nodes with and without metastases was assessed. The accuracy of miR-200a was 84.2% (95% CI, 68.1–93.4%), the positive predictive value was 100%, and the negative predictive value was 68.4% (95% CI, 43.5–87.3%). The accuracy of miR-200c was 92.1%, the positive and negative predictive values were of 100% and 81.2% (95% CI, 77.5–97.9%), respectively. For miR-203 and miR-205, both positive and negative predictive values, as well as the accuracy levels, were of 100% and AUC of 1.0 (Table 3).

**Table 3:** **Accuracy characteristics of microRNAs in discriminating metastatic and non-metastatic lymph nodes in FFPE and FNA biopsies from lymph node samples**

**microRNA**	**PPV**	**NPV**	**Accuracy**	**AUC (95% CI)**
	**% (95% CI)**	**% (95% CI)**	**% (95% CI)**	
**FFPE samples**				
**miR-200a**	100 (82.2–100.0)	68.4 (43.5–87.3)	84.2 (68.1–93.4)	0.92 (0.83–0.99)
**miR-200c**	100 (84.4–100.0)	81.2 (54.34–95.73)	92.1 (77.5–97.9)	0.94 (0.85–1.0)
**miR-203**	100 (86.2–100)	100 (75.1–100)	100 (88.6–100)	1.0 (0–1.0)
**miR-205**	100 (86.2–100)	100 (75.1–100)	100 (88.6–100)	1.0 (0–1.0)
**FNA samples classified by cytology**				
**miR-203**	100 (91.5–100)	100 (94.9–100)	100 (96.05–100)	1.0 (0–1.0)
**miR-205**	100 (91.5–100)	100 (94.9–100)	100 (96.05–100)	1.0 (0–1.0)
**FNA samples classified by histology**				
**miR-203**	100 (90.9–100)	95.9 (88.6–99.1)	97.3 (92.1–99.4)	0.963 (0.921–1.0)
**miR-205**	100 (93.2–100)	94.6 (85.1–98.8)	96.7 (93.1–100)	0.966 (0.921–1.0)

FFPE, Formalin-fixed paraffin embedded; FNA, Fine-needle aspiration; PPV, Positive predictive value; NPV, Negative predictive value; AUC, Area under the ROC curve; CI, Confidence interval.

Since miR-203 and miR-205 showed a high accuracy in identifying metastatic lymph nodes, the ability of these markers in detecting metastatic cells in a background of lymphoid tissue was also evaluated. The presence of these microRNAs was examined in the leftover material removed from the step-sectioned lymph nodes during IHC analyses. According to immunostaining, these samples harbored macrometastases (n = 5), micrometastases (n = 4), and isolated tumor cells (n = 2). As shown in Additional file 2: Figure S2, expression levels above the cutoff value could be detected in all lymph nodes carrying metastatic cells, while the expression in the non-metastatic samples was always bellow the cutoff. These results suggested that the evaluation of miR-203 and miR-205 expression still presents high sensitivity and specificity even when the metastatic deposit is ‘diluted’ in a background of lymphoid tissue.

### Validation and diagnostic accuracy of miR-203 and miR-205 expression in FNA samples

Given the high accuracy of miR-203 and miR-205 in detecting metastases in FFPE lymph node samples, we decided to evaluate these markers in FNA biopsies of lymph nodes from HNSCC patients.

**Figure 6:**
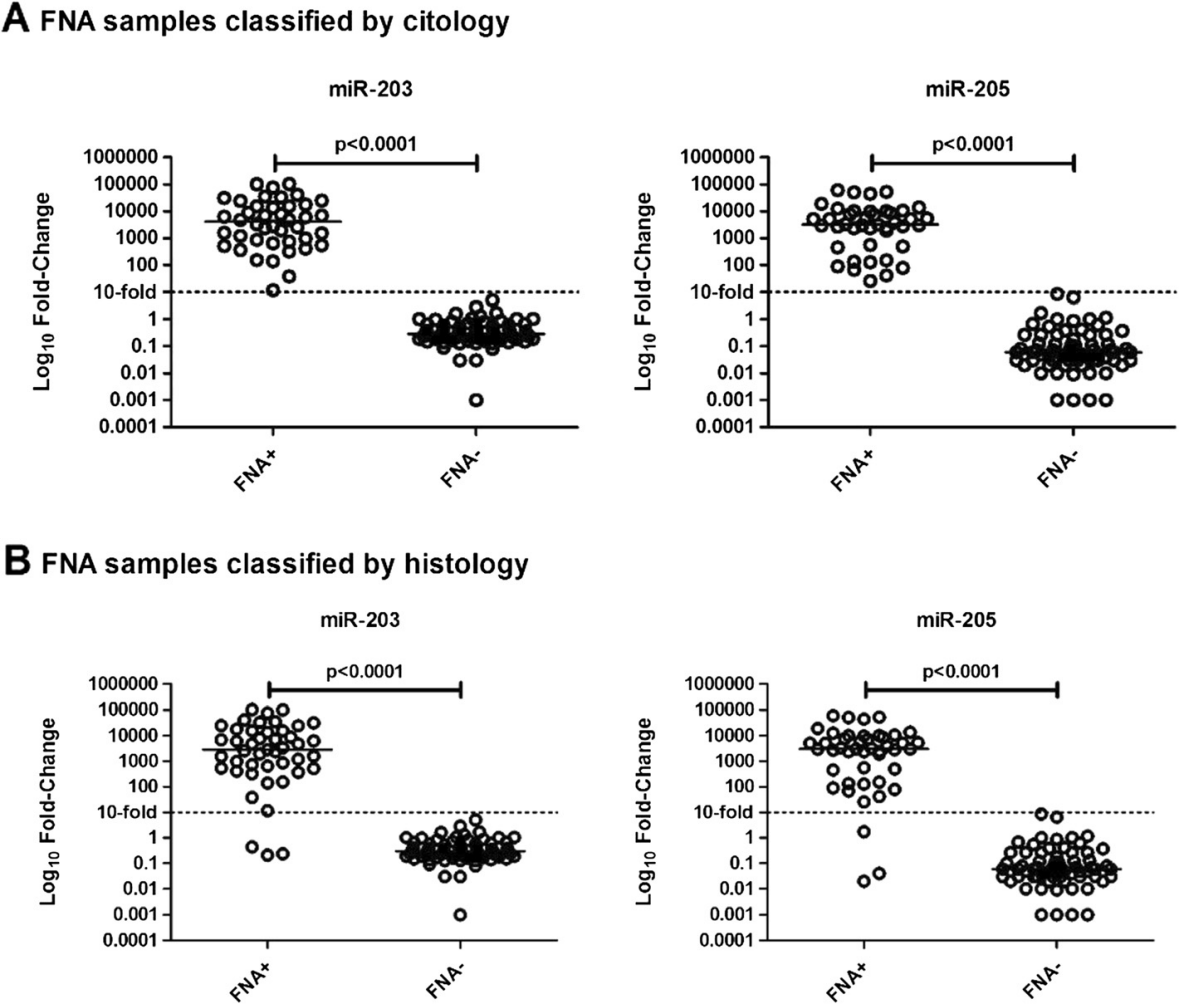
Expression profile of miR-203 and miR-205 in FNA biopsies from lymph nodes classified as positive (FNA+) or negative (FNA–) according to **(A)** cytological diagnostic (FNA+: n = 42; FNA–; n = 71) and **(B)** histological diagnostic (FNA+: n = 45; FNA–; n = 68). The Y-axis shows the log_10_ fold-change of the relative expression (2^-ΔΔCt^). The *P* value (Mann–Whitney) from each comparison is provided. The dotted line indicates the 10-fold cutoff adopted.

**Figure 7:**
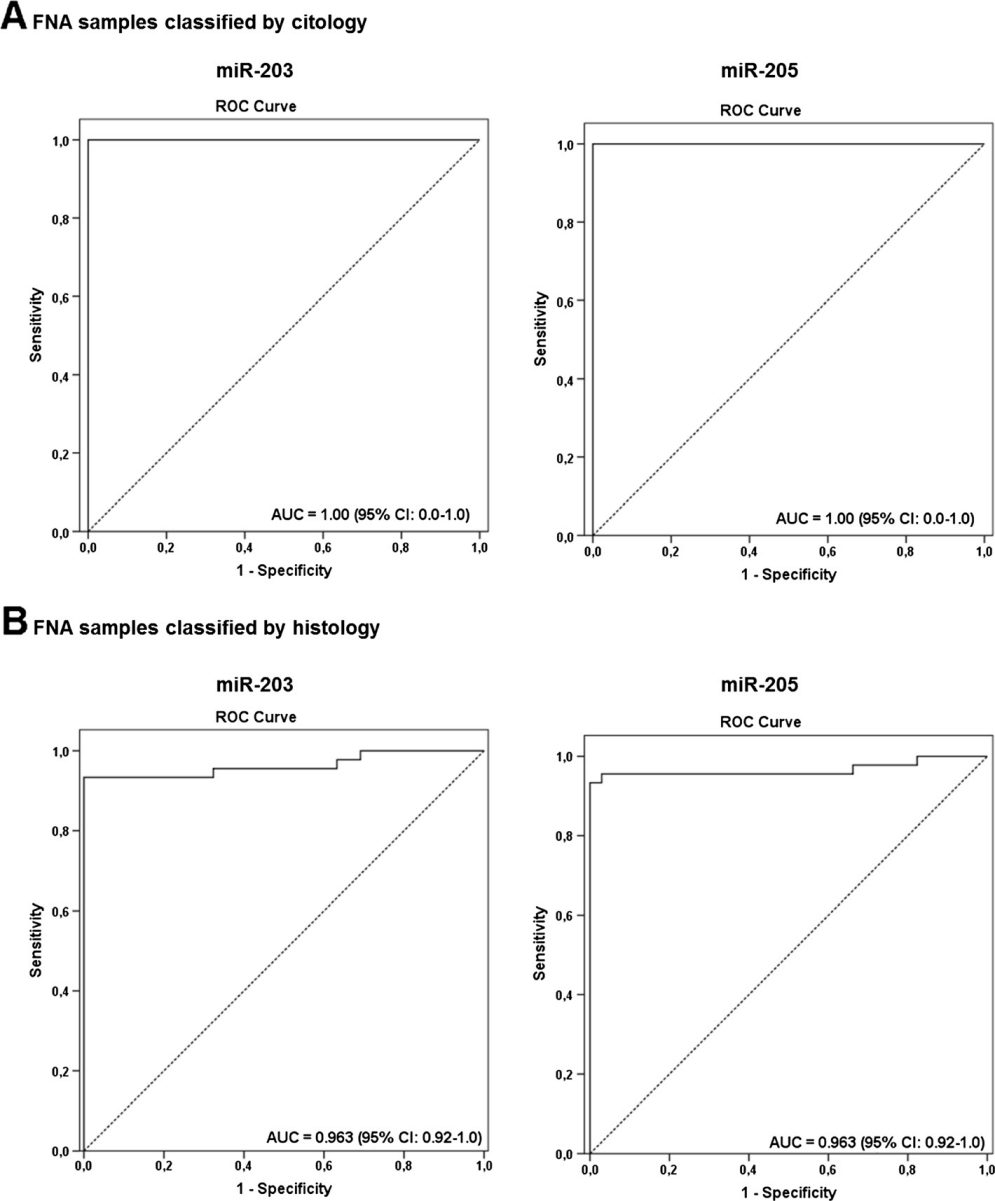
Receiver Operating Characteristic curves obtained for microRNAs miR-203 and miR-205 in in FNA biopsies from lymph nodes classified as positive (FNA+) or negative (FNA–) according to **(A)** cytological diagnostic and **(B)** histological diagnostic.

The accuracy of both markers in detecting metastatic deposits in the FNA biopsies was compared with the results obtained from cytological assessment, which had detected 42 positive FNA samples and 71 negative ones for the presence of epithelial cells. This analysis showed a complete concordance between the cytological and molecular approaches in the detection of positive and negative nodes with a Kappa value of 1.000. All 42 positive and 71 negative FNA on the cytological assessment were correctly identified by both markers with a sensitivity rate of 100% (42/42, CI 95%, 91.5–100) and a specificity level of 100% (71/71, CI 95%, 94.9–100) (Table 2; Figure 6A). A high accuracy of both miR-203 and miR-205 was observed with an AUC of 1.00 (Table 3; Figure 7A), negative and positive predictive values of 100% (95% CI, 94.9–100 and 95% CI, 91.5–100, respectively), and accuracy levels of 100% (95% CI, 96.05–100) (Table 3).

Next, the results obtained from the histological evaluation (after lymph node excision), which is considered the definitive classification of lymph nodes according to the metastatic status, were compared with the molecular analyses of the FNA samples using both miR-203 and miR-205. The histological analyses found 45 positive FNA and 68 negative biopsies. There was an agreement of 100% between both approaches in detecting negative lymph nodes and of 93.3% in classifying positive ones (Kappa value = 0.944). Only three metastatic samples (6.7%) were not detected in FNA samples using the molecular markers, the same ones harboring micrometastases and isolated tumor cells identified only in the H&E sections (after lymph node excision) and not detected by cytological assessment. This result suggests that small clusters of cells cannot be represented during the aspiration, which would preclude its detection through cytological and molecular methods. All in all, the sensitivity rate for both markers was 92.9% (39/42, CI 95%, 80.5–98.4), with a specificity level of 100% (71/71, CI 95%, 94.9–100) (Table 2; Figure 6B). A high accuracy of both miR-203 and miR-205 in distinguishing positive and negative FNA with AUC of 0.963 and 0.966, respectively, and accuracy levels of 97.3% (95% CI, 92.1–99.4) were also observed (Table 3; Figure 7B). Moreover, negative predictive values were of 95.9% (95% CI, 88.6–99.1%) and positive predictive values of 100% (95% CI, 90.9–100%) for both microRNAs (Table 3).

## Discussion

HNSCC is a heterogeneous group of tumors that arises from multiple factors that alter different pathways contributing to its development and progression. The presence of lymph node metastases is determinant for prognosis and significantly reduces the effectiveness of disease control [7,9,42]. Thus, the accurate detection of lymph node metastases in HNSCC patients is of paramount importance for its correct staging and more effective treatment planning.

Although the H&E-based pathologic examination of these tumors is very sensitive for macrometastases detection, smaller metastatic deposits are difficult to identify and the correct diagnosis relies on the slide quality, operator’s proficiency, sensitivity of the method, and, often, the need for IHC. Although IHC is a more sensitive alternative, it is a laborious and time-consuming analysis that demands the investigation of serial sections from different levels of each lymph node excised from the patient. Therefore, even though a more detailed pathological examination of lymph nodes can provide more accurate information about metastases, this involves a long time for preparation of the specimens and a heavy workload for pathologists to examine them. Taken together, all these limitations highlight the need for new molecular markers to complement the pathological methods in the task of detecting metastatic cells in lymph nodes of HNSCC patients.

In an initial ‘discovery set’ analysis, the expression pattern of 667 microRNAs was compared between metastatic and non-metastatic lymph nodes and, after filtering steps, seven microRNAs were chosen to be examined in a FFPE ‘validation set’. From those, miR-200a and miR-200c showed high levels of specificity and sensitivity, while miR-203 and miR-205 were able to detect even small metastatic deposits such as micrometastases or isolated tumor cells. Such high accuracy levels attest the feasibility of using these markers in the clinical practice as an additional tool to help pathologists in the correct assessment of lymph node status of HNSCC patients harboring sub-clinical neck metastases.

The sentinel node biopsy is a technique of cervical lymph node staging in patients with primary tumors, allowing a more detailed histological, immunohistochemical, and molecular search for occult neck metastases. Usually, the histopathological evaluation of sentinel lymph nodes is based on immunohistochemistry analysis of serial sections, requiring a long time for the final diagnosis [43]. Consequently, the delay in the correct diagnosis of the malignancy may lead to the need of additional surgeries, increasing the risk of post-operative complications and functional problems, as well as postponing the beginning of the adjuvant treatment [44,45]. In the present study, miR-203 and miR-205 were able to detect the presence of metastatic deposits even in entire lymph node samples containing large amounts of lymphocytes and lymphoid stroma. This highlights the high sensitivity of these markers in detecting epithelial cells in a lymphoid tissue background, allowing the correct detection of metastases in entire lymph nodes and avoiding false negative results related to partial lymph node assessment. Moreover, a methodology for automated qRT-PCR-based gene expression analysis in an average time of 35 minutes has been described recently [46]. Taken collectively, these results suggest the feasibility of performing microRNA marker analysis in sentinel lymph nodes in a time consistent with an intraoperative evaluation, which can be a relevant tool to assist the surgeon in deciding the best approach to be adopted in the neck treatment.

The biopsy based on cytological analysis of FNA samples is a minimally invasive method of high accuracy for the diagnosis of lesions in various organs. Its diagnostic accuracy depends on several factors, such as site, type of injury, the experience of the professional collecting the sample, the quality of the preparation, and the diagnostic skills of the cytopathologist [47,48]. This study showed that the comparison between cytology assessment, histology examination, and molecular analysis presented high levels of agreement in detecting metastatic cells in lymph nodes. Both cytology and molecular assays could not detect the presence of tumor cells in three FNA samples containing micrometastases or isolated tumor cells. We believe that the misclassification of these three false-negative samples was due to sampling errors represented by the absence of metastatic cells in the FNA specimen. Since metastatic cells were not aspirated, they were not present in the cytological smear and consequently were also absent in the sample obtained from washing the aspiration needle. Apart from those, all FFPE samples harboring micrometastases (according to pathological evaluation) could be correctly identified through the use of these molecular markers. Moreover, it is important to note that 30.4% of the FNA samples were collected from patients that have received previous radiotherapy treatment on the neck. Thus, the effects of radiation on lymph node tissues (hence, presence of necrosis and keratin granuloma) did not hinder the correct identification of metastatic cells on these samples. Even though the molecular approach did not show an improvement in accuracy when compared to the cytological assessment, we believe that its use is warranted given that, unlike molecular assessment, the accuracy of cytological assessments is highly dependent on the quality of the preparation and the diagnostic skills of the cytopathologist. The results obtained with the analysis of FNA samples indicate a high specificity and sensitivity of miR-203 and miR-205 to detect the presence of metastatic cells in the FNA biopsy samples, suggesting their role in a future pre-treatment or follow-up test of suspicious lymph nodes in HNSCC patients.

## Conclusions

To the best of our knowledge, this is the first study to demonstrate the usefulness of miR-203 and miR-205 expression as a sensitive, specific, and accurate molecular approach for the diagnosis of cervical lymph node metastases. This molecular approach can be used i) at diagnosis, by analyzing samples collected by FNA biopsies, ii) in post-surgery analyses, as described in this work, as a tool to assist the histopathological examination, allowing the investigation of entire lymph nodes and reducing sampling bias, iii) during surgery as an alternative tool for examining sentinel lymph nodes, and iv) during patient follow-up for the testing of suspicious lymph nodes, again, evaluating FNA biopsies.

Our results suggest that the evaluation of miR-203 and miR-205 expression could be an important tool for the management of HNSCC patients, assisting in the stratification of patients that may harbor neck metastases, aiding in therapy planning and patient surveillance, ultimately contributing to an improvement in quality of life and survival rates.

## Additional files

Additional file 1: Table S1. Clinical features of the 48 HNSCC patients enrolled in the FFPE validation data set – FFPE lymph nodes from patients with cervical metastases and with negative lymph nodes were evaluated. **Table S2.** Clinical features of the 79 HNSCC patients enrolled in the FNA validation data set – 113 FNA biopsies* were collected from patients who underwent neck dissection during the resection of the primary tumor, as a salvage treatment after organ preservation protocol or for the treatment of patients who developed neck metastases during follow-up. **Table S3.** Sensitivity and specificity values for the biomarkers evaluated in lymph node metastasis samples (n = 19, 14 macrometastases and 5 micrometastases), and in non-metastatic lymph nodes (n = 5). Additional file 2: Figure S1. Expression profile of miR-628-5p, miR-758, and miR-382 in lymph node samples containing macrometastasis (Ma; n = 14) and micrometastases (Mi; n = 5), and in non-metastatic lymph nodes (NM; n = 5). The Y-axis shows the fold-change (2^-ΔΔCt^) relative expression. **Figure S2.** Expression profile of microRNAs miR-203 and miR-205 in lymph nodes containing macrometastases, micrometastases, or isolated tumor cells. The microRNAs were recovered from the metastatic cell obtained after macrodissection of five 5-mm sections or by addressing the leftover material after the processing of the entire lymph node from each case. Analysis of the entire non-metastatic (NM) lymph nodes was also included as a negative control. The Y-axis shows the average of the log_10_ fold-change relative expression value (2^-ΔΔCt^) obtained after three independent assays. The dotted line indicates the cutoff value determined according to the Youden index from ROC curves. Ma, Macrometastases; Mi, Micrometastases; ITC, Isolated tumor cells.

## Abbreviations

AUC: Area under the ROC Curve
CI: Confidence interval
FFPE: Formalin-fixed, paraffin-embedded
FNA: Fine needle aspiration
H&E: Hematoxylin and Eosin
HNSCC: Head and neck squamous cell carcinoma
IHC: Immunohistochemistry
miR: microRNA
mRNA: Messenger RNA
NPV: Negative predictive value
PCR: Polymerase chain reaction
PPV: Positive predictive value
qRT-PCR: Quantitative reverse transcription polymerase chain reaction
ROC: Receiver operating characteristic
