# Erratum: Accuracy of microRNAs as markers for the detection of neck lymph node metastases in patients with head and neck squamous cell carcinoma

**DOI:** 10.1186/s12916-015-0406-4

**Published:** 2015-07-01

**Authors:** Ana Carolina de Carvalho, Cristovam Scapulatempo-Neto, Danielle Calheiros Campelo Maia, Adriane Feijó Evangelista, Mariana Andozia Morini, André Lopes Carvalho, André Luiz Vettore

**Affiliations:** Laboratory of Cancer Molecular Biology, Department of Biological Sciences, Federal University of São Paulo, Diadema, Brazil; Department of Pathology, Barretos Cancer Hospital, Barretos, Brazil; Molecular Oncology Research Center, Barretos Cancer Hospital, Barretos, Brazil; Department of Head and Neck Surgery, Barretos Cancer Hospital, Barretos, Brazil; Laboratory of Cancer Molecular Biology, Department of Biological Sciences, Federal University of São Paulo, Diadema, Brazil; Cancer and Stem Cell Biology Program, Duke-NUS Graduate Medical School, Singapore, Singapore; Departamento de Cirurgia de Cabeça e Pescoço, Hospital de Câncer de Barretos, Rua Antenor Duarte Villela, 1331, Barretos, SP 14784-400 Brazil; Laboratório de Biologia Molecular do Câncer, UNIFESP, Rua Pedro de Toledo, 669 - 11° andar, L11B, São Paulo, SP 04039-032 Brazil

## Author´s correction note

Upon reviewing our recently published research article [1], we noticed that some of the data presented in the manuscript and on the tables were incorrectly presented. These mistakes do not change the overall conclusions regarding the high accuracy of miR-203 and miR-205 as diagnostic markers of neck metastases in HNSCC.

## Corrected text

### 1. (Page 8: End of “Validation and diagnostic accuracy of miR-203 and miR-205 expression in FNA samples”)

#### Please replace

All in all, the sensitivity rate for both markers was 92.9 % (39/42, CI 95 %, 80.5–98.4), with a specificity level of 100 % (71/71, CI 95 %, 94.9–100) (Table 2; Figure 6B).

**Table 2:** Sensitivity and specificity values of microRNAs evaluated in discriminating metastatic and non-metastatic lymph nodes in FFPE and FNA biopsies from lymph node samples

microRNA		Sensitivity				Specificity
	Cutoff^a^	Metastatic^b^	Macrometastases	Micrometastases	Isolated tumor cells	Non-metastatic
		% (95 % CI) (n)	% (95 % CI) (n)	% (95 % CI) (n)	% (95 % CI) (n)	% (95 % CI) (n)
**FFPE samples**						
** miR-200a**	5.96	76.0 (54.5-89.8) (19/25)	94.4 (70.6-99.7) (17/18)	40.0 (7.3-82.9) (2/5)	0 (0–80.2) (0/2)	100 (71.7-100) (13/13)
** miR-200c**	2.33	88.0 (67.7-96.8) (22/25)	100 (78.1-100) (17/18)	80.0 (29.9-98.9) (4/5)	0 (0–80.2) (0/2)	100 (71.7-100)) (13/13)
** miR-203**	1.96	100 (83.4-100) (25/25)	100 (78.1-100) (17/18)	100 (46.3-100) (5/5)	100 (19.8-100) (2/2)	100 (71.7-100) (13/13)
** miR-205**	1.54	100 (83.4-100) (25/25)	100 (78.1-100) (17/18)	100 (46.3-100) (5/5)	100 (19.8-100) (2/2)	100 (71.7-100) (13/13)
**FNA samples classified by cytology**						
** miR-203**	10	100 (91.5-100) (42/42)	100 (91.5-100) (42/42)	N/A	N/A	100 (94.9-100) (71/71)
** miR-205**	10	100 (91.5-100) (42/42)	100 (91.5-100) (42/42)	N/A	N/A	100 (94.9-100) (71/71)
**FNA samples classified by histology**						
** miR-203**	10	92.9 (80.5-98.4) (68/71)	100 (89.3-100) (68/68)	0 (0) (0/2)	0 (0) (0/1)	100 (94.9-100) (71/71)
** miR-205**	10	92.9 (80.5-98.4) (68/71)	100 (89.3-100) (68/68)	0 (0) (0/2)	0 (0) (0/1)	100 (94.9-100) (71/71)

FFPE, formalin-fixed paraffin embedded; FNA, fine-needle aspiration; PPV, positive predictive value; NPV, negative predictive value; AUC, area under the ROC curve; CI, confidence interval; N/A, not applicable, FFPE, formalin-fixed paraffin embedded; FNA, fine-needle aspiration; PPV, positive predictive value; NPV, negative predictive value; AUC, area under the ROC curve; CI, confidence interval

^a^the cutoff values for FFPE samples were determined according to the Youden index (value in which the difference between sensitivity and 1-specificity is maximum) obtained from the ROC curves

^b^the "metastatic" group comprises all cases with positive lymph nodes (macrometastases, micrometastases or isolated tumor cells)

#### With the amended text

All in all, the sensitivity rate for both markers was 93.3 % (42/45, CI 95 %, 81.7–98.6), with a specificity level of 100 % (68/68, CI 95 %, 94.7–100) (Table 2; Figure 6B).

**Table 2:** Sensitivity and specificity values of microRNAs evaluated in discriminating metastatic and non-metastatic lymph nodes in FFPE and FNA biopsies from lymph node samples

microRNA		Sensitivity				Specificity
	Cutoff^a^	Metastatic^b^	Macrometastases	Micrometastases	Isolated tumor cells	Non-metastatic
		% (95 % CI) (n)	% (95 % CI) (n)	% (95 % CI) (n)	% (95 % CI) (n)	% (95 % CI) (n)
**FFPE samples**						
** miR-200a**	5.96	76.0 (54.5-89.8) (19/25)	94.4 (70.6-99.7) (17/18)	40.0 (7.3-82.9) (2/5)	0 (0–80.2) (0/2)	100 (71.7-100) (13/13)
** miR-200c**	2.33	88.0 (67.7-96.8) (22/25)	100 (78.1-100) (18/18)	80.0 (29.9-98.9) (4/5)	0 (0–80.2) (0/2)	100 (71.7-100)) (13/13)
** miR-203**	1.96	100 (83.4-100) (25/25)	100 (78.1-100) (18/18)	100 (46.3-100) (5/5)	100 (19.8-100) (2/2)	100 (71.7-100) (13/13)
** miR-205**	1.54	100 (83.4-100) (25/25)	100 (78.1-100) (18/18)	100 (46.3-100) (5/5)	100 (19.8-100) (2/2)	100 (71.7-100) (13/13)
**FNA samples classified by cytology**						
** miR-203**	10	100 (91.5-100) (42/42)	100 (91.5-100) (42/42)	N/A	N/A	100 (94.9-100) (71/71)
** miR-205**	10	100 (91.5-100) (42/42)	100 (91.5-100) (42/42)	N/A	N/A	100 (94.9-100) (71/71)
**FNA samples classified by histology**						
** miR-203**	10	93.3 (81.7-98.6) (42/45)	100 (91.5-100) (42/42)	0 (0) (0/2)	0 (0) (0/1)	100 (94.7-100) (68/68)
** miR-205**	10	93.3 (81.7-98.6) (42/45)	100 (91.5-100) (42/42)	0 (0) (0/2)	0 (0) (0/1)	100 (94.7-100) (68/68)

FFPE, formalin-fixed paraffin embedded; FNA, fine-needle aspiration; PPV, positive predictive value; NPV, negative predictive value; AUC, area under the ROC curve; CI, confidence interval; N/A, not applicable, FFPE, formalin-fixed paraffin embedded; FNA, fine-needle aspiration; PPV, positive predictive value; NPV, negative predictive value; AUC, area under the ROC curve; CI, confidence interval

^a^the cutoff values for FFPE samples were determined according to the Youden index (value in which the difference between sensitivity and 1-specificity is maximum) obtained from the ROC curves

^b^the "metastatic" group comprises all cases with positive lymph nodes (macrometastases, micrometastases or isolated tumor cells)

### 2. (Page 8: End of “Validation and diagnostic accuracy of miR-203 and miR-205 expression in FNA samples”)

#### Please replace

Moreover, negative predictive values were of 95.9 % (95 % CI, 88.6–99.1 %) and positive predictive values of 100 % (95 % CI, 90.9–100 %) for both microRNAs (Table 3).

**Table 3:** Accuracy characteristics of microRNAs in discriminating metastatic and non-metastatic lymph nodes in FFPE and FNA biopsies from lymph node samples

microRNA	PPV	NPV	Accuracy	AUC (95 % CI)
	% (95 % CI)	% (95 % CI)	% (95 % CI)	
**FFPE samples**				
** miR-200a**	100 (82.2-100.0)	68.4 (43.5-87.3)	84.2 (68.1-93.4)	0.92 (0.83-0.99)
** miR-200c**	100 (84.4-100.0)	81.2 (54.34-95.73)	92.1 (77.5-97.9)	0.94 (0.85-1.0)
** miR-203**	100 (86.2-100)	100 (75.1-100)	100 (88.6-100)	1.0 (0–1.0)
** miR-205**	100 (86.2-100)	100 (75.1-100)	100 (88.6-100)	1.0 (0–1.0)
**FNA samples classified by cytology**				
** miR-203**	100 (91.5-100)	100 (94.9-100)	100 (96.05-100)	1.0 (0–1.0)
** miR-205**	100 (91.5-100)	100 (94.9-100)	100 (96.05-100)	1.0 (0–1.0)
**FNA samples classified by histology**				
** miR-203**	100 (90.9-100)	95.9 (88.6-99.1)	97.3 (92.1-99.4)	0.963 (0.921-1.0)
** miR-205**	100 (93.2-100)	94.6 (85.1-98.8)	96.7 (93.1-100)	0.966 (0.921-1.0)

FFPE, formalin-fixed paraffin embedded; FNA, fine-needle aspiration; PPV, positive predictive value; NPV, negative predictive value; AUC, area under the ROC curve; CI, confidence interval

#### With the amended text

Moreover, negative predictive values were of 95.9 % (95 % CI, 88.6–99.1 %) and positive predictive values of 100% (95 % CI, 91.5–100 %) for both microRNAs (Table 3).

**Table 3:** Accuracy characteristics of microRNAs in discriminating metastatic and non-metastatic lymph nodes in FFPE and FNA biopsies from lymph node samples

microRNA	PPV	NPV	Accuracy	AUC (95 % CI)
	% (95 % CI)	% (95 % CI)	% (95 % CI)	
**FFPE samples**				
** miR-200a**	100 (82.2-100.0)	68.4 (43.5-87.3)	84.2 (68.1-93.4)	0.92 (0.83-0.99)
** miR-200c**	100 (84.4-100.0)	81.2 (54.34-95.73)	92.1 (77.5-97.9)	0.94 (0.85-1.0)
** miR-203**	100 (86.2-100)	100 (75.1-100)	100 (88.6-100)	1.0 (0–1.0)
** miR-205**	100 (86.2-100)	100 (75.1-100)	100 (88.6-100)	1.0 (0–1.0)
**FNA samples classified by cytology**				
** miR-203**	100 (91.5-100)	100 (94.9-100)	100 (96.05-100)	1.0 (0–1.0)
** miR-205**	100 (91.5-100)	100 (94.9-100)	100 (96.05-100)	1.0 (0–1.0)
**FNA samples classified by histology**				
** miR-203**	100 (91.5-100)	95.9 (88.6-99.1)	97.3 (92.1-99.4)	0.963 (0.921-1.0)
** miR-205**	100 (91.5-100)	95.9 (88.6-99.1)	97.3 (92.1-99.4)	0.966 (0.921-1.0)

FFPE, formalin-fixed paraffin embedded; FNA, fine-needle aspiration; PPV, positive predictive value; NPV, negative predictive value; AUC, area under the ROC curve; CI, confidence interval
