# Supplementary material for: Accuracy of microRNAs as markers for the detection of neck lymph node metastases in patients with head and neck squamous cell carcinoma
Source: BMC Med. 2015 May 9;13:108. doi: 10.1186/s12916-015-0350-3 (PMC4493814; doi:10.1186/s12916-015-0350-3)
Supplement: Additional file 1: Table S1. — Clinical features of the 48 HNSCC patients enrolled in the FFPE validation data set – FFPE lymph nodes from patients with cervical metastases and with negative lymph nodes were evaluated. Table S2. Clinical features of the 79 HNSCC patients enrolled in the FNA validation data set – 113 FNA biopsies* were collected from patients who underwent neck dissection during the resection of the primary tumor, as a salvage treatment after organ preservation protocol or for the treatment of patients who developed neck metastases during follow-up. Table S3. Sensitivity and specificity values for the biomarkers evaluated in lymph node metastasis samples (n = 19, 14 macrometastases and 5 micrometastases), and in non-metastatic lymph nodes (n = 5). [file 12916_2015_350_MOESM1_ESM.docx]

**Additional file 1**

**Table S1:** Clinical features of the 48 HNSCC patients enrolled in the FFPE validation data set – FFPE lymph nodes from patients with cervical metastases and with negative lymph nodes were evaluated.

| **Feature** | **Category** | **n** | **%** |
| --- | --- | --- | --- |
| **Age** | Mean | 59.3 |  |
|  | Median | 60 |  |
|  | Range | 43-84 |  |
|  |  |  |  |
| **Gender** | Male | 38 | 79.2 |
|  | Female | 10 | 20.8 |
|  |  |  |  |
| **Tobacco Consumption** | Yes | 23 | 47.9 |
|  | No | 21 | 43.8 |
|  | N/A | 4 | 8.3 |
|  |  |  |  |
| **Tumor site** | Floor of mouth | 15 | 31.3 |
|  | Lower gum | 1 | 2.1 |
|  | Oral tongue | 28 | 58.3 |
|  | Alveolar ridge | 4 | 8.3 |
|  |  |  |  |
| **cT** | cT1 | 10 | 20.8 |
|  | cT2 | 30 | 62.5 |
|  | cT3 | 8 | 16.7 |
|  |  |  |  |
| **Adjuvant radiotherapy** | No | 21 | 43.8 |
|  | Yes | 23 | 47.9 |
|  | N/A | 4 | 8.3 |
|  |  |  |  |
| **pN status^1^** | macrometastases | 18 | 37.5 |
|  | micrometastases | 5 | 10.4 |
|  | isolated tumor cells | 2 | 4.2 |
|  | No | 23 | 47.9 |
|  |  |  |  |
| **Recurrence** | No | 27 | 56.3 |
|  | Local | 7 | 14.6 |
|  | Regional | 6 | 12.5 |
|  | Lung metastases | 3 | 6.2 |
|  | N/A | 5 | 10.4 |
|  |  |  |  |
| **Status** | Alive; disease-free | 28 | 58.3 |
|  | Alive; with cancer | 3 | 6.2 |
|  | Deceased by other cases | 1 | 2.1 |
|  | Deceased by cancer | 14 | 29.2 |
|  | N/A | 2 | 4.2 |

^1^Histopathology evaluation comprises H&E and IHC for cytokeratins. N/A: cases in which the variable values were not available.

**Table S2:** Clinical features of the 79 HNSCC patients enrolled in the FNA validation data set – 113 FNA biopsies^*^ were collected from patients who underwent neck dissection during the resection of the primary tumor, as a salvage treatment after organ preservation protocol or for the treatment of patients who developed neck metastases during follow-up.

| **Feature** | **Category** | **n** | **%** |
| --- | --- | --- | --- |
| **Age** | Mean | 57.35 |  |
|  | Median | 57.0 |  |
|  | Range | 29-78 |  |
|  |  |  |  |
| **Gender** | Male | 70 | 88.6 |
|  | Female | 9 | 11.4 |
|  |  |  |  |
| **Tobacco Consumption** | Yes | 57 | 76.0 |
|  | No | 18 | 24.0 |
|  |  |  |  |
| **Tumor site** | Oral cavity | 47 | 59.5 |
|  | Larynx | 12 | 15.2 |
|  | Oropharynx | 15 | 19.0 |
|  | Hypopharynx | 5 | 6.3 |
|  |  |  |  |
| **Tumor Stage** | Initial (I/II) | 17 | 21.5 |
|  | Advanced (III/IV) | 62 | 78.5 |
|  |  |  |  |
| **FNA biopsy colection** | at the resection of the primary tumor | 55 | 69.6 |
|  | at salvage treatment | 19 | 24.1 |
|  | at treatment of neck metastases | 5 | 6.3 |
|  |  |  |  |
| **FNA Status by Histology** | positive | 45 | 39.8 |
|  | negative | 68 | 60.2 |
|  |  |  |  |
| **FNA Status by Cytology** | positive | 42 | 37.2 |
|  | negative | 71 | 62.8 |
|  |  |  |  |
| **Status** | Alive; disease-free | 57 | 72.2 |
|  | Alive; with cancer | 15 | 19.0 |
|  | Deceased by other causes | 1 | 1.3 |
|  | Deceased by cancer | 6 | 7.6 |

^* Whenever possible, more than one FNA biopsies were collected from the same patient accounting for 113 FNA biopsies collected^

**Table S3:** Sensitivity and specificity values for the biomarkers evaluated in lymph node metastasis samples (n=19, 14 macrometastases and 5 micrometastases), and in non-metastatic lymph nodes (n=5)

| **microRNA** | **Sensitivity** | | | **Specificity** |
| --- | --- | --- | --- | --- |
|  | **Metastatic**^1^ **% (n)** | **Macrometastases % (n)** | **Micrometastases % (n)** | **Non-metastatic % (n)** |
| **miR-628-5p** | 26,3 (5) | 35,7 (5) | 0 (0) | 100 (0) |
| **miR-758** | 31,6 (6) | 42,8 (6) | 0 (0) | 100 (0) |
| **miR-382** | 52,6 (10) | 64,3 (9) | 20 (1) | 100 (0) |
| **miR-200a** | 84,2 (16) | 100 (14) | 40 (2) | 100 (0) |
| **miR-200c** | 94,7 (18) | 100 (14) | 80 (4) | 100 (0) |
| **miR-203** | 100 (19) | 100 (14) | 100 (5) | 100 (0) |
| **miR-205** | 100 (19) | 100 (14) | 100 (5) | 100 (0) |

^1^the "metastatic" group comprises all cases with positive nodes, whether they are micro or macrometastases
