# Supplementary material for: Accuracy of microRNAs as markers for the detection of neck lymph node metastases in patients with head and neck squamous cell carcinoma
Source: BMC Med. 2015 May 9;13:108. doi: 10.1186/s12916-015-0350-3 (PMC4493814; doi:10.1186/s12916-015-0350-3)
Supplement: Additional file 2: Figure S1. — Expression profile of miR-628-5p, miR-758, and miR-382 in lymph node samples containing macrometastasis (Ma; n = 14) and micrometastases (Mi; n = 5), and in non-metastatic lymph nodes (NM; n = 5). The Y-axis shows the fold-change (2-ΔΔCt) relative expression. Figure S2. Expression profile of microRNAs miR-203 and miR-205 in lymph nodes containing macrometastases, micrometastases, or isolated tumor cells. The microRNAs were recovered from the metastatic cell obtained after macrodissection of five 5-mm sections or by addressing the leftover material after the processing of the entire lymph node from each case. Analysis of the entire non-metastatic (NM) lymph nodes was also included as a negative control. The Y-axis shows the average of the log10 fold-change relative expression value (2-ΔΔCt) obtained after three independent assays. The dotted line indicates the cutoff value determined according to the Youden index from ROC curves. Ma, Macrometastases; Mi, Micrometastases; ITC, Isolated tumor cells. [file 12916_2015_350_MOESM2_ESM.docx]

**Additional file 2**

**
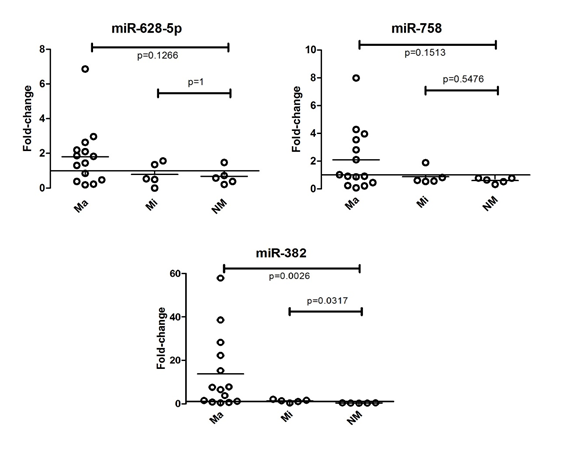
**

**Figure S1:** Expression profile of miR-628-5p, miR-758 and miR‑382 in lymph node samples containing macrometastasis (Ma; n=14), micrometastases (Mi; n=5) and in non-metastatic lymph nodes (NM; n=5). The Y-axis shows the fold-change (2^-ΔΔCt^) relative expression.

**
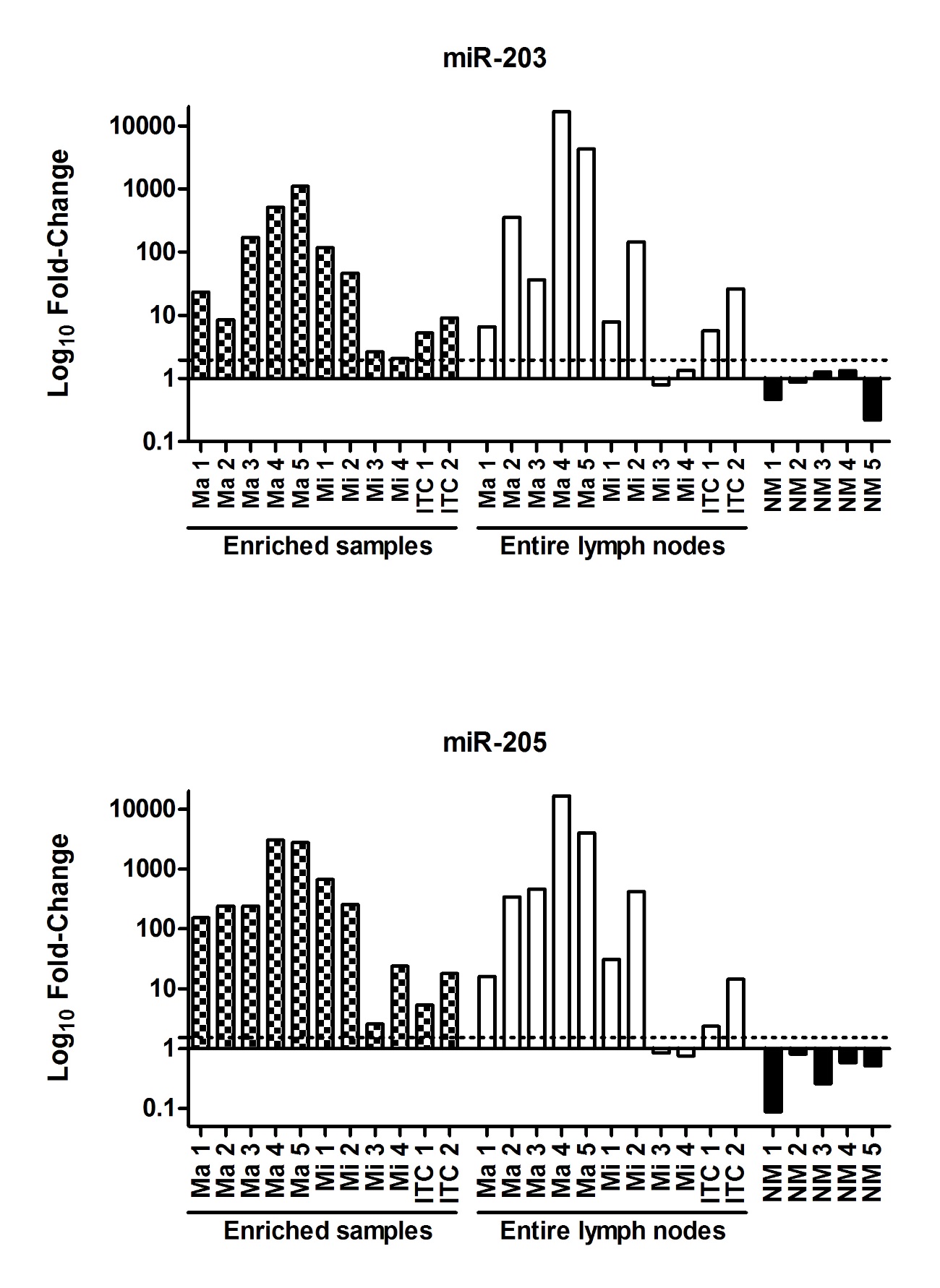
**

**Figure S2:** Expression profile of microRNAs miR-203 and miR-205 in lymph nodes containing macrometastases, micrometastases or isolated tumor cells. The microRNAs were recovered from the metastatic cell obtained after macrodissection of five 5-mm sections or by addressing the leftover material after the processing of the entire lymph node from each case. The analysis of entire non-metastatic lymph nodes (NM) was also included as negative control. The Y-axis shows the average of the log_10_ fold-change relative expression value (2^-ΔΔCt^) obtained after three independent assays. The dotted line indicates the cutoff value determined according to the Youden index from ROC curves. Ma (macrometastases), Mi (micrometastases), ITC (isolated tumor cells).
